# Supplementary material for: The origin of septin ring size control in budding yeast
Source: EMBO J. 2025 Oct 2;44(22):6466–98. doi: 10.1038/s44318-025-00571-5 (PMC12623784; doi:10.1038/s44318-025-00571-5)
Supplement: Supplementary file 1 — Appendix [file 44318_2025_571_MOESM1_ESM.pdf]

Appendix for "The origin of septin ring size control in budding yeast"

Table of Contents

Supplementary Information ……………………………………………………………….2

Appendix Figure S1 ……………………………………………………………….4

Appendix Figure S2 ……………………………………………………………….6

Appendix Figure S3 ……………………………………………………………….8

Appendix Figure S4 ……………………………………………………………..…10

Appendix Figure S5 ……………………………………………………….........11

Supplementary Information

Septin ring formation model based on previously described principles is not robust to parameter changes

Our results suggest that exocytosis is diffused in *bni1*Δ. Since exocytosis displaces proteins from the polarity site in *S. pombe* [Gerganova et al., 2021] and has been proposed to sculpt the septin ring in *S. cerevisiae* by displacing septin [Okada et al., 2013], we hypothesized that diffused exocytosis could produce a larger ring diameter by septin displacement (Fig. 4h). To evaluate the plausibility of this, we turned to computational modeling.

Okada et al. [Okada et al., 2013] showed via computational modeling that exocytosis can sculpt a septin ring. Based on this suggested mechanism, we implemented a model. However, even though this model was able to form a ring, it was not robust to changes in model parameters including cell volume. Additionally, more recent studies [Lai et al., 2018] have shown that septin ring formation can start in the absence of a Sec4 signal (exocytosis marker), suggesting additional mechanisms in addition to exocytosis-promoting ring formation.

Therefore, we implemented a new model (Fig. EV4a,b). The precise recruitment mechanism of septin is unknown, but septin monomers interact with polarity proteins Gic1/2, Axl2, and Cdc24 [Iwase et al., 2006; Chollet et al., 2020; Kang et al., 2024], hence we model that Axl2 (representing the other polarity factors) recruits septin. Cdc24 has been observed to bind Cdc11 in the Cdc42-GTP cluster, but not in the septin ring [Chollet et al., 2020]. To achieve ring formation without exocytosis, we model that polarity proteins (here Axl2) bind to septin and inhibit polymerization within the Cdc42 cluster area, subsequently forcing polymerization at the cluster periphery (Fig. EV4b). Lastly, following Okada et al. [Okada et al., 2013], exocytosis is directed towards the pole where it displaces proteins, and septin recruits GAP proteins (Fig. EV4b).

Because septin recruitment is likely cell-cycle triggered [Lai et al., 2018], and to reduce simulation time (one simulation takes > 100 hours), we initiated simulations from a Cdc42-GTP cluster (Fig. EV4c). As observed experimentally [Okada et al., 2013], also in the model Cdc42-GTP concentration initially decreased, and then increased when a stable ring formed. In the Okada et al. [Okada et al., 2013] model this was partially achieved by having septin act as a diffusion barrier. Interestingly, in our model without any diffusion barrier, the septin-recruited GAP achieved a similar effect by trapping and concentrating Cdc42-GTPs within the ring (Fig. EV4c).

Next, we investigated if diffused exocytosis produced a larger ring diameter. Starting from the same Cdc42-GTP cluster we simulated 10 cells for two model parameter combinations. Exocytosis was modeled to be directed towards the Cdc42 cluster, and as we use a finite element method (FEM) simulator where the model's geometry is meshed, we simulated concentrated and diffused exocytosis by allowing vesicles to hit approximately 0.03% or 0.34% of the mesh nodes respectively. Using a custom ring diameter computing algorithm, we found that the septin ring diameter was unaffected by diffused exocytosis (Fig. EV4d,e).

The lack of effect of diffused exocytosis on ring diameter can depend on model parameter values. Parameters are tuned to yield realistic dynamics, however, due to simulation runtime (a single run takes >100 hours) an exhaustive search is infeasible. Thus, we created a simple fast-to-simulate particle model that captures key properties of ring formation. This includes that septin particles are recruited into a cluster area (representing the Cdc42-GTP cluster), and that the septin particles diffuse on the membrane, recycle, and are displaced by exocytosis

(Appendix Fig. S3). Surprisingly, for different model parameters, diffused exocytosis did not produce a large septin ring diameter (Appendix Fig. S3). Similar results hold for a simpler particle simulator, where non-moving particles are displaced solely by exocytosis (Appendix Fig. S3).

In summary, the septin ring formation model suggested in Okada et al. [Okada et al., 2013] is not robust to changes in cell volume, while our model partially based on the mechanism suggested in Okada et al. does not explain why diffused exocytosis leads to an increase in septin ring diameter.

Appendix Figure S1

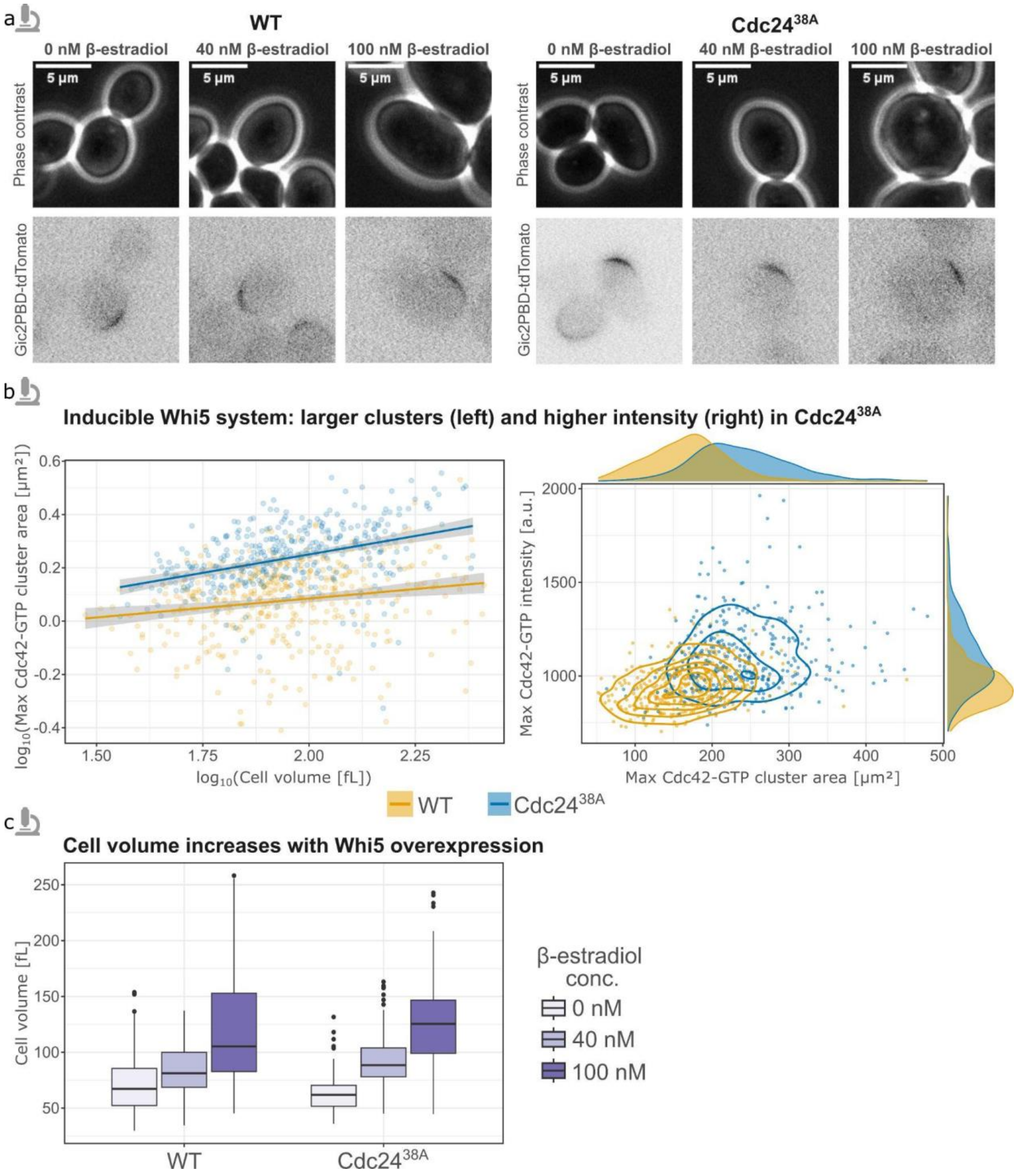

Appendix Figure S1. Cdc42-GTP cluster scaling and intensity in strains with hormone inducible Whi5. (a) Representative microscopy images of budding yeast cells (phase contrast) and Cdc42-GTP clusters (Gic2PBD-tdTomato) for wild-type Cdc24 (WT) and Cdc24^{38A} cells. (b) Quantification of microscopy results. (left) Maximum Cdc42-GTP cluster area plotted against corresponding cell volume in double logarithmic scale. Solid lines show linear regression fits. (right) Maximum Cdc42-GTP intensity in the cluster at the time when the cluster reaches its maximum area, plotted against the corresponding Cdc42-GTP cluster area. (c) Cell volume for wild-type Cdc24 and Cdc24^{38A} cells for different β-estradiol concentrations inducing Whi5 expression. In each plot, wild-type: 0 nM β-estradiol n = 123, 40 nM β-estradiol n = 111, 100 nM β-estradiol n = 181; Cdc24^{38A}: 0 nM β-estradiol n = 122, 40 nM β-estradiol n = 99, 100 nM β-estradiol n = 145. For boxplots, the center line indicates the median; box limits show the 25th-75th percentiles (IQR); whiskers extend to the most extreme data points within

1.5× IQR; points represent outliers. Two independent replicates were performed for the experiments. Microscope icon - experimental results.

Appendix Figure S2

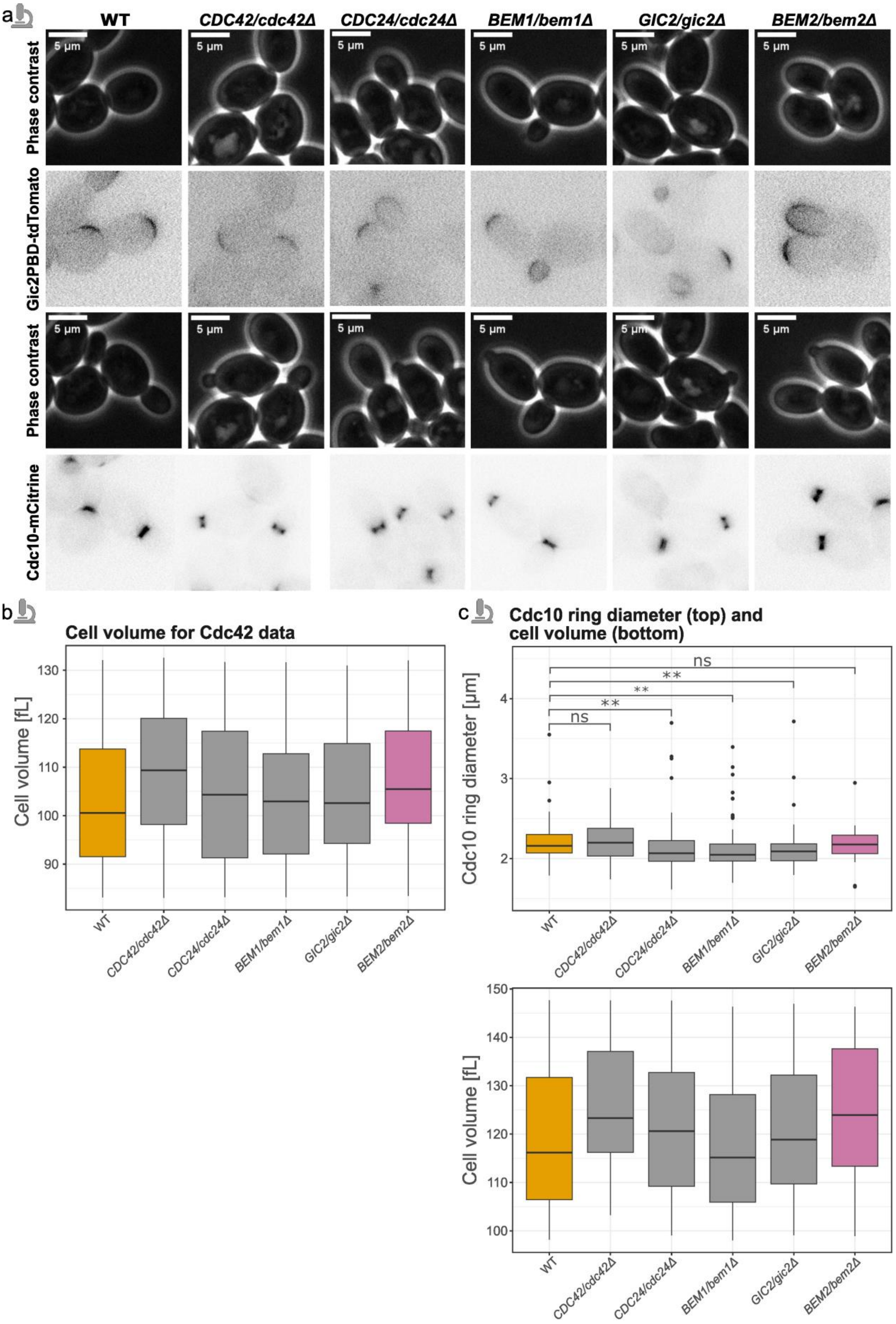

Appendix Figure S2. Experimental results for *CDC42*, *CDC24*, *BEM1*, *GIC2*, *BEM2* hemizygote mutants and WT. (a) Representative microscopy images for all stains tested

(images for WT and BEM2/bem2Δ also shown in Fig. 2G and Fig. 6C). (b) Cell volumes corresponding to Cdc42-GTP cluster area quantification shown in Fig. 2g: WT (n = 198), CDC42/cdc42Δ (n = 52), CDC24/cdc24Δ (n = 131), BEM1/bem1Δ (n = 147), GIC2/gic2Δ (n = 100), BEM2/bem2Δ (n = 54) (c) Quantification of septin ring diameter (top) and corresponding cell volume (bottom) for all tested strains: WT (n = 136), CDC42/cdc42Δ (n = 46), CDC24/cdc24Δ (n = 122), BEM1/bem1Δ (n = 108), GIC2/gic2Δ (n = 89), BEM2/bem2Δ (n = 43). For boxplots, the center line indicates the median; box limits show the 25th-75th percentiles (IQR); whiskers extend to the most extreme data points within 1.5× IQR; points represent outliers. Stars denote p-values *>0.05, **>0.0005, ***>5e-6 from Wilcoxon rank sum test. At least two independent replicates were performed for the experiments. Microscope icon - experimental results.

Appendix Figure S3

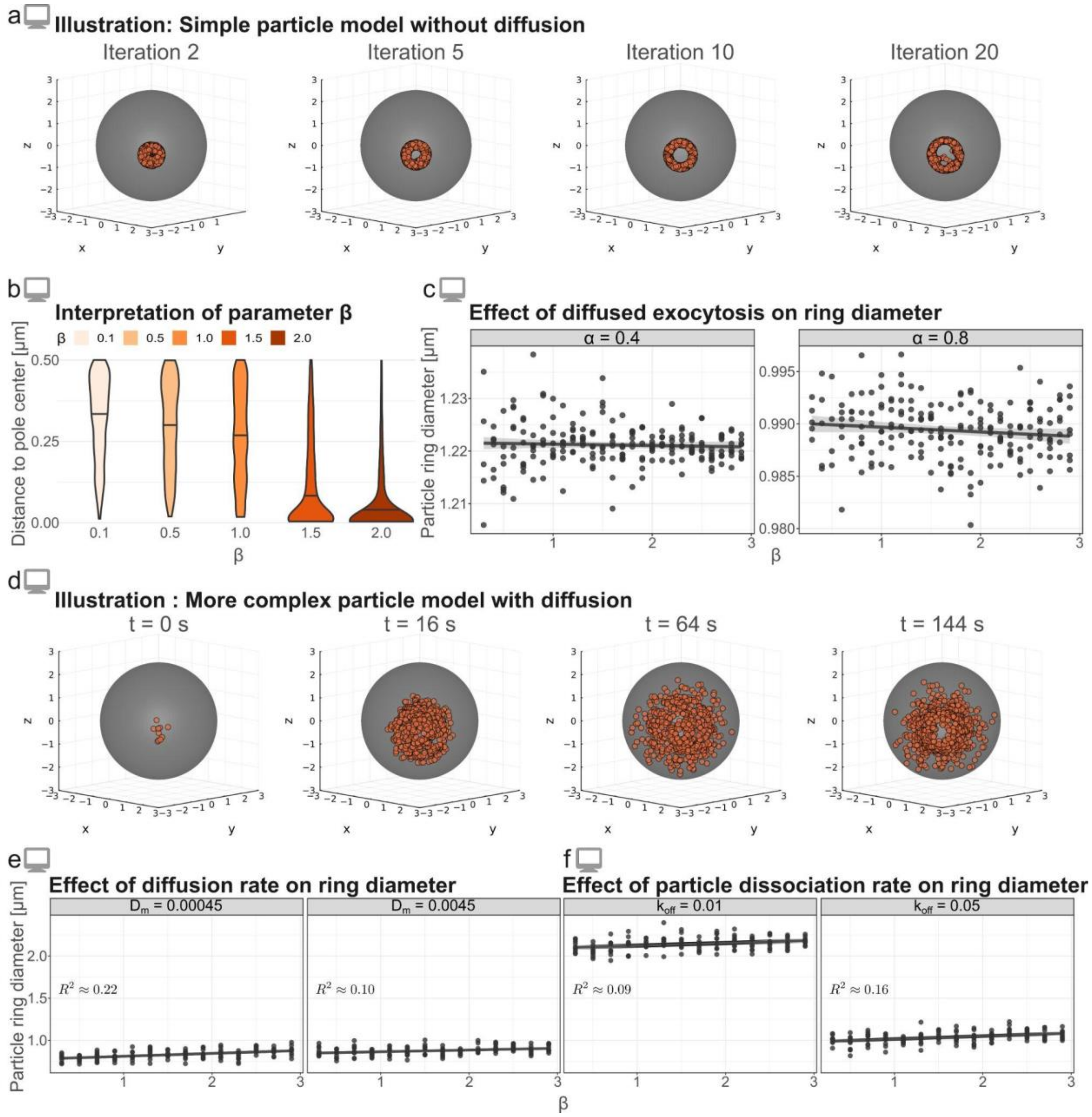

Appendix Figure S3. Particle models suggest that diffused exocytosis itself does not produce a larger septin ring diameter. (a) Illustration of the simple particle model without diffusion. Simulations start with particles covering 3% of a circular cluster area (red), followed by 20 iterations of exocytosis. (b) Parameter β affects the spread of exocytosis. Exocytosis can occur in the same circular cluster area covering 3% of the surface that particles start in or are recruited into. For a large β, exocytosis is concentrated, as seen by the distribution of the distance to the cluster area from where exocytosis occurs. Meanwhile, for a smaller β, exocytosis is more diffused. (c) Septin ring diameter as a function of β for different values of the exocytosis model parameter α (see methods). Each plot shows n=210 simulations. (d) Illustration of a more complex particle model than in contrast to the earlier particle model also includes diffusion and particle recruitment, where particles are recruited into a cluster occupying 3% of the area, and then diffuse at rate D_m, disassociate at rate k_{off}, and undergo exocytosis. (e-f) Septin ring diameter as a function of β for the second particle model, shown for different values of the diffusion rate D_m (e) and the dissociation rate k_{off} (f). Low diffusion rates and high dissociation rates lead to larger rings with concentrated exocytosis. Each plot

shows n=140 simulations. (c,e-f) Solid lines show linear regression fits. Computer icon - modeling results.

Appendix Figure S4

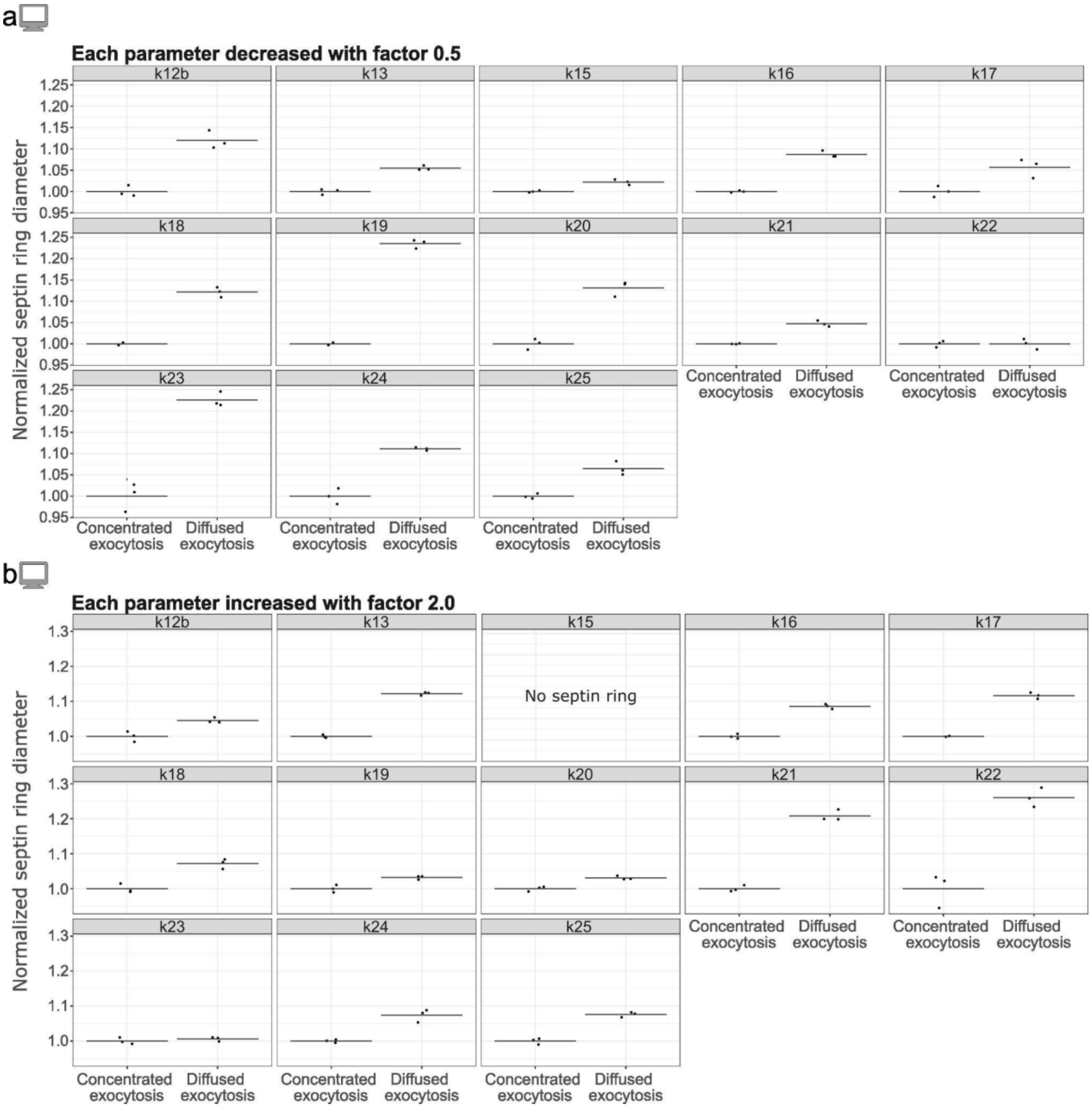

Appendix Figure S4. Sensitivity analysis for the SBER model. Normalized model ring diameter with respect to the concentrated exocytosis case when perturbing each septin-module parameter by a factor of 0.5 (a) or 2.0 (b). A septin ring failed to form in panel (a) for k15. The reaction each parameter is associated with can be found in Tab. 4. k12b sets the dissociation rate of septin-recruited GAPs from the membrane; k13 the hydrolysis strength of septin-recruited GAPs; k15 the dissociation strength of septin monomers from polarity proteins; k16 and k18 the reaction strengths of septin polymerization; k19 and k20 the binding strengths of septin monomers to polarity proteins (with k20 corresponding to cytosolic septin binding); k21 and k22 the dissociation strengths from the membrane of septin and septin-bound polarity factors; k23 the recruitment strength of polarity proteins that recruit septin; k24 the dissociation strength from the membrane of the vesicle-delivered septin-recruiting protein; and k25 the recruitment strength of septin via this vesicle-delivered protein. The line shows the average value of three simulations. Computer icon - modeling results.

Appendix Figure S5

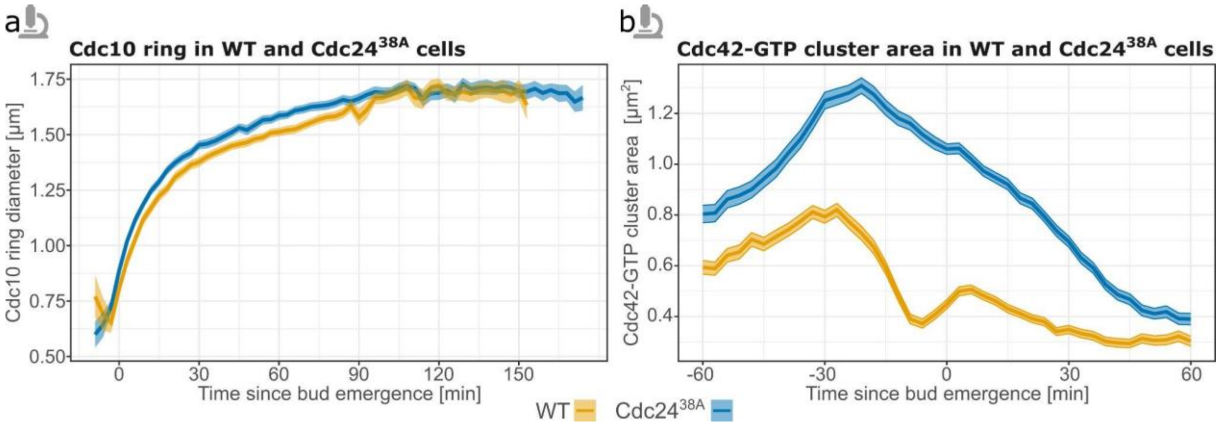

Appendix Figure S5. Septin ring and Cdc42-GTP cluster formation in negative feedback mutant. (a) Mean Cdc10 ring diameter measurements from microscopy images plotted over the cell cycle for WT (n = 68 cells) and Cdc24^{38A} (n = 101 cells). (b) Mean Cdc42-GTP cluster area measurements from microscopy images plotted over the cell cycle for WT (n = 453 cells) and Cdc24^{38A} (n = 465 cells). Single-cell traces are aligned at bud emergence. The ribbons correspond to the standard error. Two independent replicates were performed for the experiments. Microscope icon - experimental results.
