# Supplementary material for: The origin of septin ring size control in budding yeast
Source: EMBO J. 2025 Oct 2;44(22):6466–98. doi: 10.1038/s44318-025-00571-5 (PMC12623784; doi:10.1038/s44318-025-00571-5)
Supplement: Supplementary file 9 — Expanded View Figures [file 44318_2025_571_MOESM9_ESM.pdf]

Expanded View Figures

Figure EV1. Alternative computational model of Cdc42 polarization with positive feedback and increased GAP activity and protein dilution in the positive feedback model. ▶

(**A**) Schematic drawing of the alternative model with positive feedback from [Borgqvist et al, 2021] (left), and result from a representative simulation (right). (**B**) Cdc42-GTP cluster area measured at steady-state (after long simulation time) for the model plotted against cell volume in a double logarithmic scale. In each case, $n = 11$ cells with volumes ranging from 115 to 345 fL were simulated starting from random initial conditions. Note that the volume interval differs from Fig. 1 as the model polarizes in a different parameter regime compared to the models in Fig. 1. (**C**) Cdc42-GTP maximum concentration against cell volume for the same simulated cells as in (**B**), measured at the same time point as the cluster area. Solid lines in (**B**, **C**) show linear regression fits. (**D**, **E**) Cdc42-GTP cluster area (**D**) and Cdc42-GTP concentrations (**E**) for the positive feedback model measured at steady-state (after long simulation time) plotted against cell volume in a double logarithmic scale for normal (1) and stronger (1.8) GAP activity. The increased GAP activity mimics negative feedback by reducing Cdc42 activation. In each case, $n = 60$ (three replicates per volume) cells with volumes ranging from 65 to 270 fL were simulated starting from random initial conditions. Solid lines show loess smoothings. (**F**) Protein slope as in Fig. 2, but obtained from cell size mutants (see [Lanz et al, 2024] for more details). Bars show the mean value of $n = 3$ biological replicates. Computer icon—modeling results. Microscope icon—experimental results.

 © The Author(s)

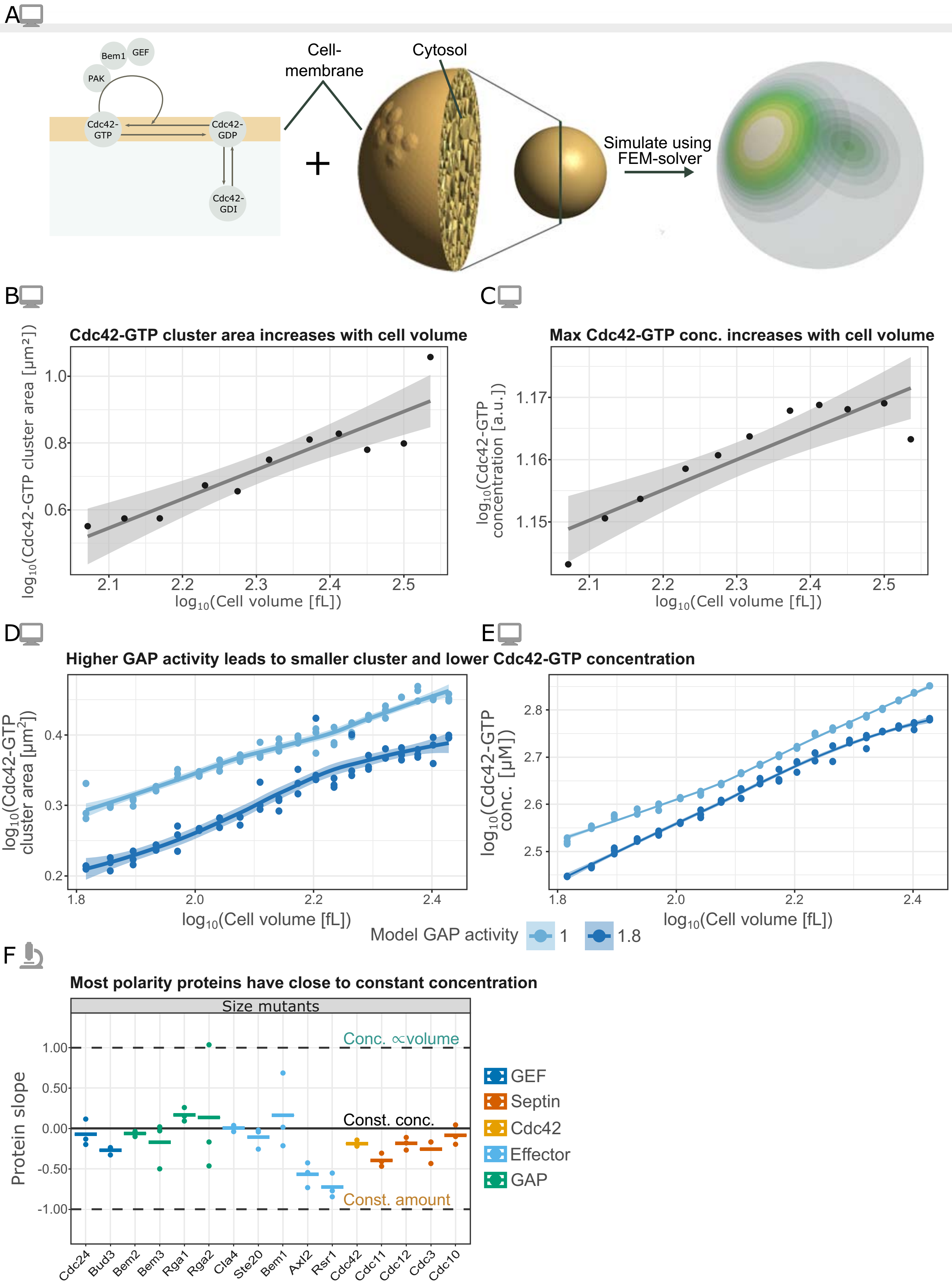
A
Bem1
GEF
PAK
Cdc42-GTP
Cdc42-GDP
Cdc42-GDI
Cell-membrane
Cytosol
Simulate using FEM-solver
B
Cdc42-GTP cluster area increases with cell volume
log10(Cdc42-GTP cluster area [µm²])
log10(Cell volume [fL])
C
Max Cdc42-GTP conc. increases with cell volume
log10(Cdc42-GTP concentration [a.u.])
log10(Cell volume [fL])
D
E
Higher GAP activity leads to smaller cluster and lower Cdc42-GTP concentration
log10(Cdc42-GTP cluster area [µm²])
log10(Cdc42-GTP conc. [µM])
log10(Cell volume [fL])
Model GAP activity 1 1.8
F
Most polarity proteins have close to constant concentration
Size mutants
Conc. ∝volume
Const. conc.
Const. amount
Protein slope
Cdc24 Bud3 Bem2 Bem3 Rga1 Rga2 Cla4 Ste20 Bem1 Axl2 Rsr1 Cdc42 Cdc11 Cdc12 Cdc3 Cdc10
GEF
Septin
Cdc42
Effector
GAP

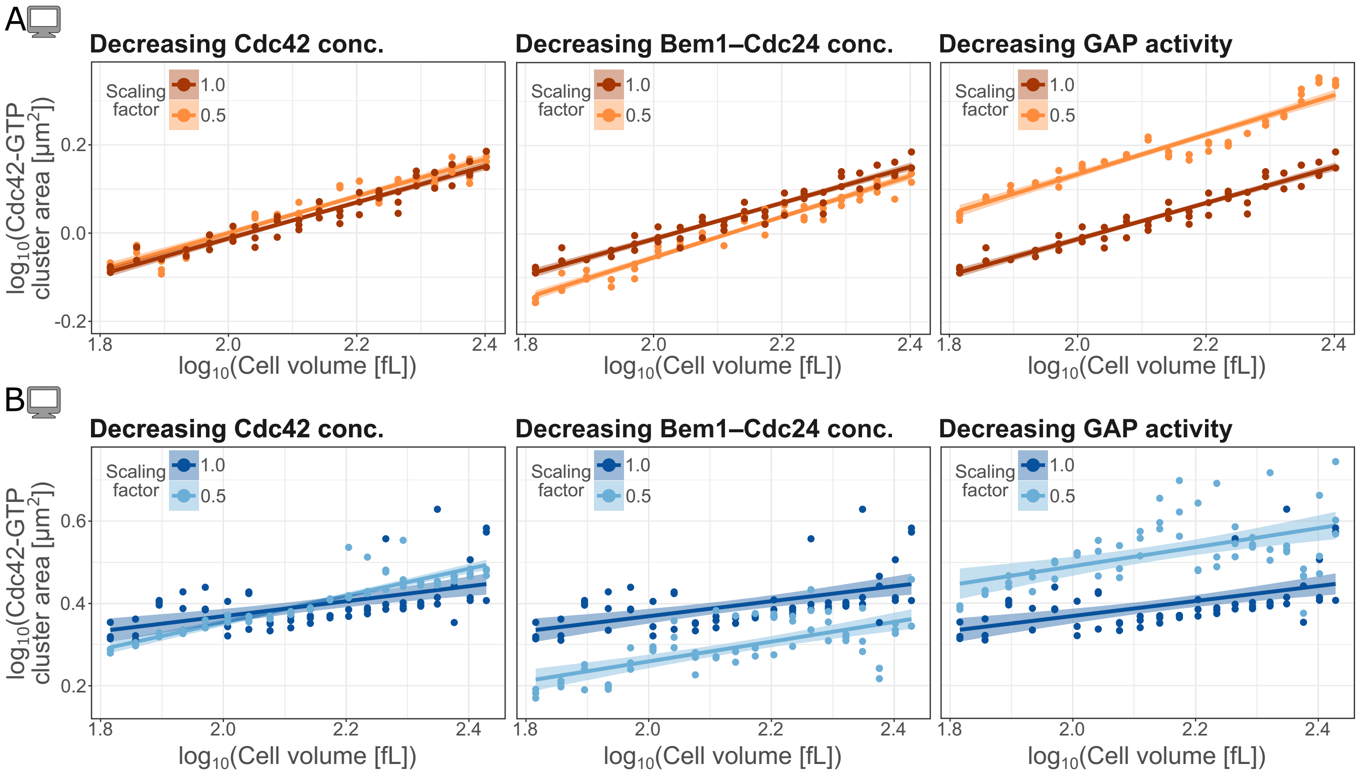

Figure EV2. Computational modeling of Cdc42 cluster area from various perturbations.

Reducing Cdc42 concentration (left), Bem1-Cdc24 concentration (middle) and GAP activity (right) for the negative feedback model (**A**) and the positive feedback model (**B**) with a factor of 0.5. In each case, for $n = 60$ (three replicates per volume), cells with volumes in the range of 65 to 270 fL were simulated starting from random initial conditions. Computer icon—modeling results.

 © The Author(s)

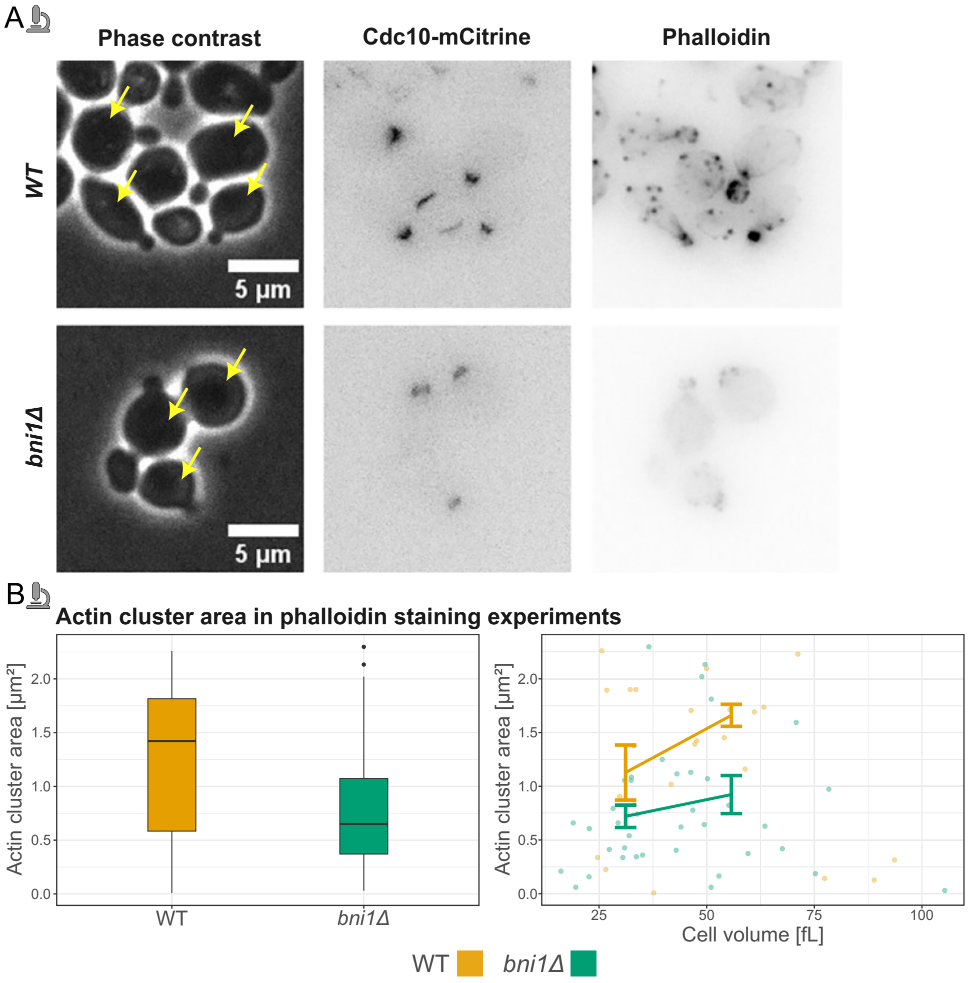

Figure EV3. Phalloidin-based measurements of F-actin show that the actin cluster area at the bud site is decreased in *bni1Δ* cells.

(**A**) Representative microscopy images of budding yeast cells (phase contrast), septin ring (Cdc10-mCitrine), and F-actin (Phalloidin) for wild-type and *bni1Δ* cells. Arrows point to cells selected for analysis. (**B**) Quantification of actin cluster area at the bud site based on phalloidin staining for wild-type ($n = 23$) and *bni1Δ* ($n = 40$) cells. Left plot: the center line indicates the median; box limits show the 25th–75th percentiles (IQR); whiskers extend to the most extreme data points within 1.5× IQR; points represent outliers. Right plot: solid lines show binned means, and error bars show standard error centered at the binned mean. Two independent replicates were performed for the experiments. Microscope icon—experimental results.

© The Author(s)

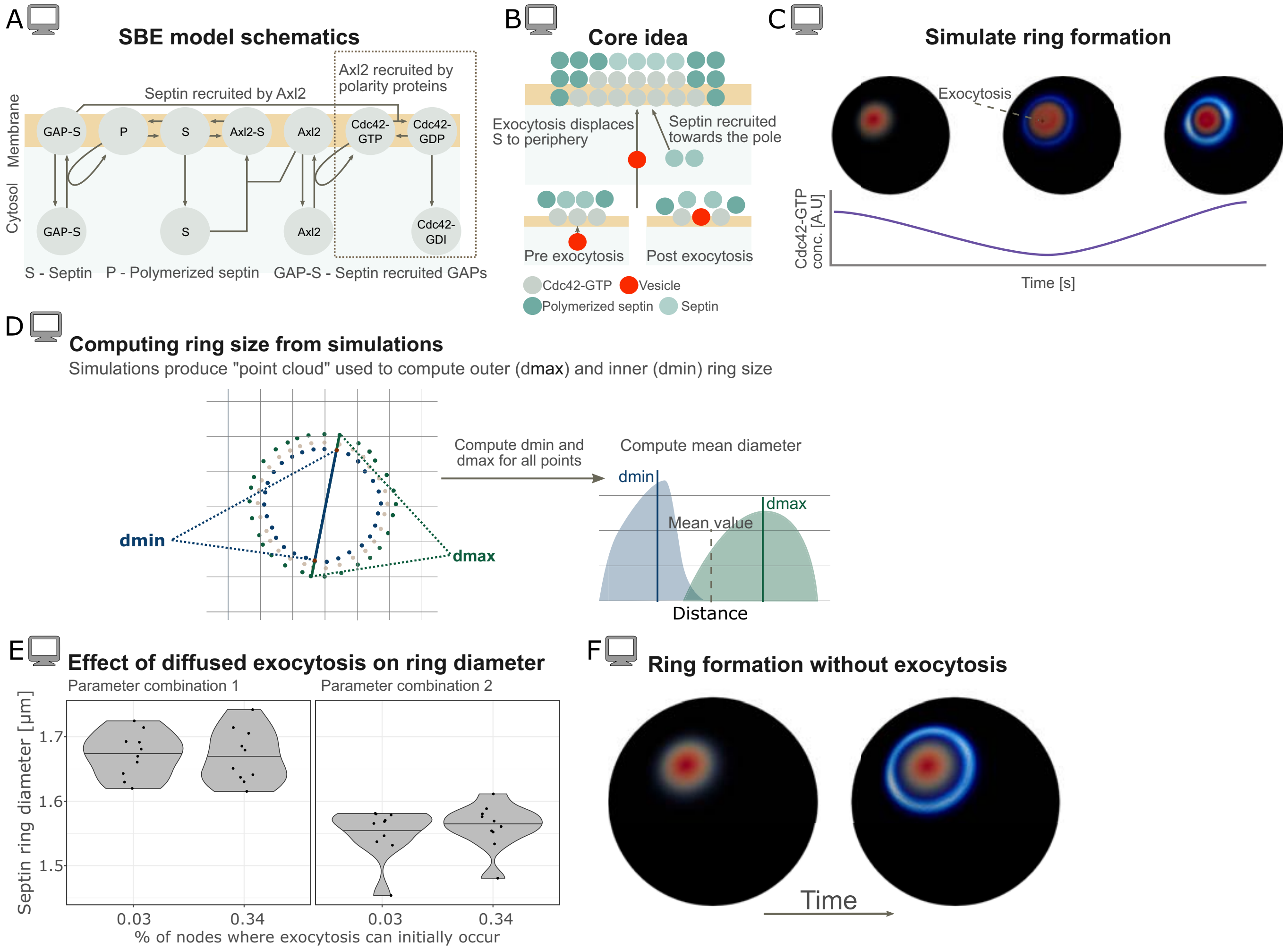

Figure EV4. The septin binding and exocytosis (SBE) model does not explain why septin ring size increases with diffused exocytosis.

Model reaction schematics (**A**) and core idea (**B**) of the SBE model. Briefly, septin is recruited by Axl2, and on the membrane, septin binds to Axl2. This binding prevents polymerization in the cluster center, which promotes polymerization and, subsequently, ring formation at the cluster periphery. Additionally, exocytosis is directed towards the cluster, which further pushes septin to the periphery. (**C**) Representative example showing Cdc42 polarization (red) and consecutive septin ring formation (blue). Cdc42-GTP concentration first decreases when septin is recruited, and then increases when a stable septin ring starts to form. (**D**) Schematic explanation of how septin ring diameter is computed from model simulations. (**E**) Septin ring diameter *(dmin+dmax)/2* (see Fig. 5) for the SBE model plotted for two parameter combinations and two levels of diffused exocytosis. For each condition, $n = 10$ simulations, all starting from the same Cdc42-GTP cluster, were performed. In each case, the model was simulated for a long time to reach a stable ring, and then the septin ring diameter was measured. The number of nodes that can be hit corresponds to nodes where the concentration of Cdc42 fulfills: *Cdc42-GTP* > *ε*max(Cdc42-GTP)*, where a smaller *ε* corresponds to more diffused exocytosis. (**F**) Example of septin ring formation for the SBER model without exocytosis. Note that without exocytosis, the SBER model is equivalent to the SBE model. Computer icon—modeling results.

 © The Author(s)

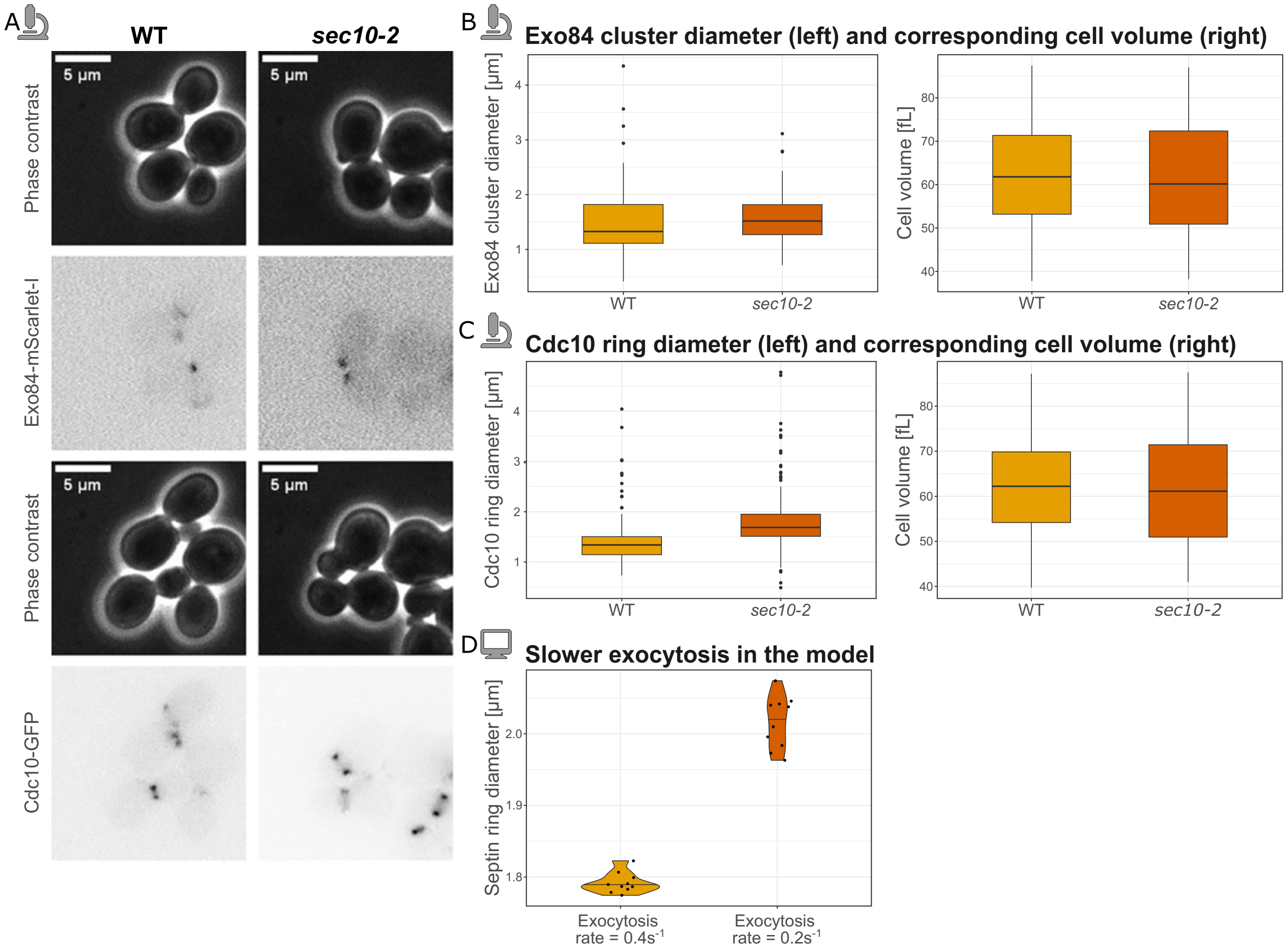

Figure EV5. Experiments with *sec10-2* temperature-sensitive mutant and reduction of exocytosis rate in the SBER model show enlarged septin rings.

Cells were analyzed between 170 and 410 min after shifting to the non-permissive temperature. (**A**) Representative microscopy images for wild-type and *sec10-2* cells. (**B**) Quantification of Exo84 cluster diameter and corresponding cell volume: WT ($n = 121$), *sec10-2* ($n = 80$). (**C**) Quantification of septin ring diameter and corresponding cell volume: WT ($n = 380$), *sec10-2* ($n = 175$). For boxplots, the center line indicates the median; box limits show the 25th–75th percentiles (IQR); whiskers extend to the most extreme data points within 1.5× IQR; points represent outliers. (**D**) Modeling results for normal (0.4 s^{-1}, $n = 10$) and reduced (0.2 s^{-1}, $n = 10$) exocytosis rate. Two independent replicates were performed for the experiments. Computer icon—modeling results. Microscope icon—experimental results.

© The Author(s)
